# Epidemiological, temporal and spatial dynamics of leprosy in a municipality in northeastern Brazil (2008-2017): an ecological study

**DOI:** 10.1590/0037-8682-0246-2020

**Published:** 2020-10-21

**Authors:** Aldenyeslle Rodrigues de Albuquerque, José Victor de Mendonça Silva, Emiliano de Oliveira Barreto, Carlos Alberto de Carvalho Fraga, Walcelia Oliveira dos Santos, Maria Salésia Moreira da Silva, Carlos Dornels Freire de Souza, Carolinne Sales-Marques

**Affiliations:** 1 Universidade Federal de Alagoas, Centro de Ciências Médicas e Enfermagem, Curso de Medicina, Arapiraca, AL, Brasil.; 2 Universidade Federal de Alagoas, Instituto de Ciências Biológicas e da Saúde, Laboratório de Biologia Celular, A/C Simões, AL, Brasil.; 3 Centro de Referência Integrado de Arapiraca, Secretaria Municipal da Saúde de Arapiraca, AL, Brasil.; 4 Universidade Federal de Alagoas, Centro de Ciências Médicas e Enfermagem, Laboratório de Biologia Molecular e Expressão Gênica, Arapiraca, AL, Brasil.

**Keywords:** Leprosy, Epidemiology, Spatio-Temporal Analysis, Arapiraca

## Abstract

**INTRODUCTION:**

Leprosy is a chronic infectious disease caused by *Mycobacterium leprae.*This study aimed to analyze the epidemiological, temporal, and spatial dynamics ofleprosy in a municipality in northeastern Brazil.

**METHODS:**

This is an ecological study on new leprosy cases in the population of Arapiraca (Alagoas, Northeast Region, Brazil), from 2008 to 2017. Data extracted from a national database were analyzed forepidemiological indicators, factors associated with physical disabilities, and spatialanalysis in the neighborhoods of Arapiraca.

**RESULTS:**

A total of 292 new cases of leprosy were recorded, particularly occurring among the following groups: women, the age group of 46-59 years, brown-skinned individuals, people with less than eight years of schooling, and urban residents; the new cases were also predominantly the tuberculoid form and were of the paucibacillary classification of the disease. Almost 1/3 of the people had some degree of physical disability, which was mainly associated with the group 60 years of age and older, black ethnicity, and the multibacillary clinical form of leprosy. The joinpoint regression showed a stationary temporal behavior of indicators. There was a heterogeneous spatial distribution with active transmission areas, especially in the neighborhoods Primavera, Baixão, Ouro Preto, and downtown.

**CONCLUSIONS:**

The epidemiological indicators revealed complexity in the process of leprosy development. These spatial and temporal studies are relevant to help in the planning, monitoring, and guidance of interventions in the municipality. The spatial analysis showed heterogeneous distribution in the analyzed neighborhoods.

## INTRODUCTION

Leprosy is a chronic contagious infectious disease caused by *Mycobacterium leprae*, an obligate intracellular alcohol-acid resistant bacillus (AARB)^1^ ^,^ ^2^ ^,^ ^3^ ^,^ ^4^. This microorganism mainly affects the skin and peripheral nerves and is of clinical and epidemiological importance mainly due to its disabling potential^5^ ^,^ ^6^ ^,^ ^7^ ^,^ ^8^.

According to the World Health Organization (WHO), 143 countries reported 214,783 new cases of leprosy in 2016, which shows a detection rate of 2.9 cases per 100 thousand inhabitants^9^. Ninety-four percent of leprosy cases were concentrated in thirteen nations around the world, and India and Brazil were in the top positions, respectively^10^.

In Brazil, 25,218 new cases of leprosy were recorded in 2016, reaching a detection rate of 12.2/100 thousand inhabitants. Of this total, 1,696 occurred in people under 15 years old^11^. Brazil has the second highest number of annual diagnoses and was the only country that did not reach the goal of eliminating the disease as a public health problem (HP) by the year 2015^10^ ^,^ ^12^.

The spatial distribution of leprosy is quite heterogeneous among the geographic regions of Brazil^13^ ^,^ ^14^ ^,^ ^15^ ^,^ ^16^. The Mid West Region (37.27/100 thousand inhabitants) had the highest general detection rate between 2012-2016, followed by the North Region (34.27/100 thousand inhabitants). The Northeast Region (23.42/10 thousand inhabitants) had the third-highest detection rate in the same period^11^.

In the northeastern states, there is also heterogeneity. In Maranhão, the prevalence was 4.91/10 thousand inhabitants in 2017, whereas, in the state of Alagoas, leprosy had a prevalence of 0.76/10 thousand inhabitants. In children under 15 years old in Alagoas, the detection rate of new cases was 2.97/100 thousand inhabitants in 2017^11^.

In the municipality of Arapiraca, where this study was conducted, 84 new cases of leprosy were recorded from 2014 to 2017. Leprosy had a general detection rate of 16.23/100 thousand inhabitants and a prevalence of 0.51/10 thousand inhabitants in Arapiraca in 2017^17^. Despite the epidemiological relevance of leprosy, studies on this disease in Alagoas’ municipalities are insufficient. Given the above, this study aimed to analyze the epidemiological, temporal, and spatial dynamics of leprosy in a municipality in northeastern Brazil from 2008 to 2017.

## METHODS

### Study area and design

**FIGURE 1:**
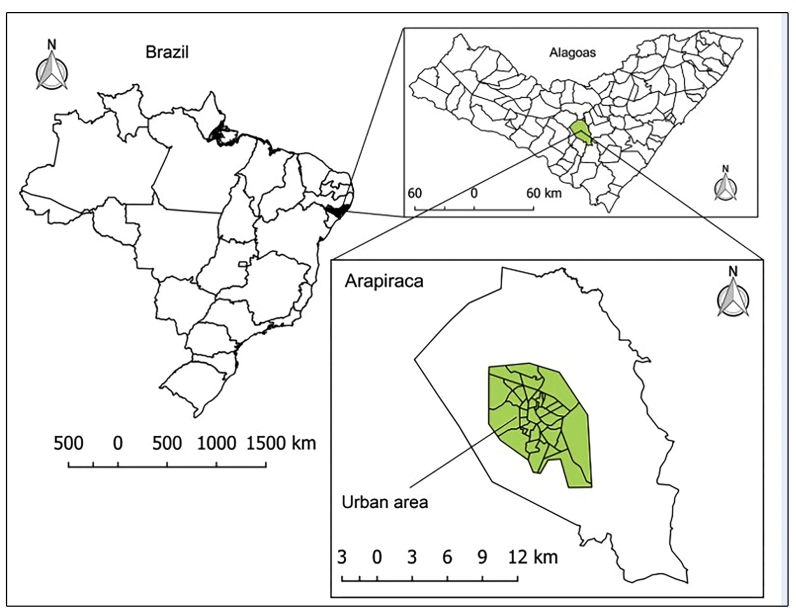
Geographic location of the study area, Arapiraca (Alagoas, Northeast Region, Brazil).

This is a mixed ecological study involving new cases of leprosy recorded from 2008 to 2017 in the population of the municipality of Arapiraca, located in the Agreste region of Alagoas. Arapiraca is the second-most populous municipality in the state of Alagoas, with an estimated population of 230,400 inhabitants in 2018. This municipality has a Human Development Index (HDI) of 0.649, which is below the national average (HDI, 0.699), an aging rate of 6.19%, a Gini Index of 0.55, a per capita income of R$423.28, and 37.80% of the population live in poverty or extreme poverty^18^ ^,^ ^19^. Arapiraca comprises thirty-eight neighborhoods, which were classified as spatial geographical units for the analysis (Figure 1).

### Source of data

Nine sociodemographic variables (gender, age group, ethnicity/color, schooling level, area of residence, clinical condition, operational classification, mode of detection, and degree of physical disability) and three epidemiological indicators (annual detection coefficient of new cases of leprosy in the general population per 100 thousand inhabitants, annual detection coefficient of new cases of leprosy in children under 15 years old per 100 thousand inhabitants, and detection coefficient of new cases of leprosy with grade 2 disability at the time of diagnosis per 1 million inhabitants) were selected for monitoring the elimination process of the disease as a public HP.

The incidence rates of cases per 100 thousand inhabitants were classified based on the Ministry of Health’s (MH) classification: low (0.00-2.00), medium (2.00-9.99), high (10.00-19,99), very high (20.00-39.99), and hyperendemic (≥40.00). For children under 15 years old, the rate of cases per 100 thousand inhabitants was classified as low (0.00-0.49), medium (0.50-2.49), high (2.50-4,99), very high (5.00-9.99), and hyperendemic (≥10.00)^20^.

The data referring to leprosy cases were obtained from the Information System for Disease Notification (SINAN) in Brazil from the Municipal Health Department of Arapiraca. Duplications and cases classified as a diagnostic error were excluded. The necessary population data were obtained from the Brazilian Institute of Geography and Statistics (IBGE).

### Data analysis

The data analysis was structured in three stages. The first one consisted of sociodemographic and clinical characterization, using simple descriptive statistics (absolute and relative frequencies), and the identification of factors associated with the presence of physical disability using the chi-square test (χ^2^) and odds ratio (OR) calculation.

In the second stage, the trend of the three selected epidemiological indicators was analyzed. For this, the joinpoint regression model was used. This model makes it possible to adjust the data of a temporal series starting from the smallest possible number of inflection points^21^. The trends were classified as increasing, decreasing, or stationary, according to the slope of the regression line. The annual percent change (APC) and average annual percent change (AAPC) were also calculated.

The third stage consisted of spatial modeling and identification of the highest risk areas of leprosy occurrence in Arapiraca. Global Moran’s I statistic was used to identify spatial autocorrelation (spatial dependence). Once identified, the Local Moran statistic (Local Index of Spatial Association [LISA]) was used to find the highest risk areas of occurrence of the disease. Using the LISA, the study neighborhoods were positioned in the Moran scattering diagram: Q1 = High/high (positive values and positive means), Q2 = Low/low (negative values and negative means), Q3 = high/low (positive values and negative means), and Q4 = low/high (negative values and positive means). The neighborhoods located in the first quadrant (Q1) were considered a priority^22^. In addition, the relative risk in the neighborhoods was calculated considering the municipal rate as a reference. Then, thematic maps were made to present the results.

A 5% significance level and a 95% confidence interval were considered in all analyses. The following software was used: i) Statistical Package Software for the Social Sciences, version 22.0 (SPSS, Inc., Chicago, IL, USA); ii) Joinpoint regression program, version 4.5.0.1 (Statistical Research and Applications Branch, National Cancer Institute, Rockville, MD, USA); and iii) QGis, version 2.14.11 (Open Source Geospatial Foundation - OSGeo, Beaverton, OR, USA).

### Ethical considerations

The study was approved by the institutional ethics committee (Research Ethics Committee of the Federal University of Alagoas; approval no. 3,213,273, March 21, 2019). 

## RESULTS

Sociodemographic and clinical characterization and factors associated with the presence of physical disabilities

Between 2008 and 2017, 292 new leprosy cases were recorded in Arapiraca (detection rate of 17.02/100 thousand). Regarding the sociodemographic characteristics, there was a preponderance of women (54.1%; n = 151) and individuals in the age group of 46-59 years (29.5%; n = 86); 3.8% (n = 11) of individuals were under 15 years old (detection rate of 2.38/100 thousand), 68.8% were brown-skinned (n = 201), and 65.4% had less than 8 years of schooling (n = 191). As for the area of residence, 88% of patients (n = 257) lived in the urban area of Arapiraca. With regard to the clinical variables, the tuberculoid clinical form (38.7%; n = 113) and the paucibacillary (PB) operational classification (54.3%; n = 156) stood out, and referral (83.2%; n = 243) was the main mode of detecting leprosy new cases (Table 1).

**TABLE 1:** Sociodemographic and epidemiological characterization of new leprosy cases diagnosed in Arapiraca (Alagoas, Northeast Region, Brazil), 2008-2017.

Variable	n	%
**Gender**		
Female	158	54.1
Male	134	45.9
**Age group**		
<15	11	3.8
15-30	66	22.6
31-45	86	29.4
46-59	72	24.7
60 and over	57	19.5
**Race/color**		
White	53	18.2
Black	24	8.2
Brown-skinned	201	68.8
Indigenous	1	0.3
Unknown	13	4.5
**Schooling**		
Illiterate	54	18.5
1-8 years	137	46.9
≥9 years	57	19.5
Unknown	44	15.1
**Area of residence**		
Urban	257	88.0
Rural	35	12.0
Clinical form		
Undetermined	41	14.0
Tuberculoid	113	38.7
Dimorphic	78	26.7
Virchowian	40	13.7
Unclassified	20	6.9
**Operational Classification**		
Multibacillary	136	46.6
Paucibacillary	156	53.4
**Detection mode**		
Referrals	243	83.2
Spontaneous demand	24	8.2
Collective examination	1	0.3
Examination of contacts	20	6.9
Others/Unknown	4	1.4
**Degree of physical disability**		
Grade 0	151	51.7
Grade 1	74	25.3
Grade 2	18	6.2
Unclassified	49	16.8

Almost a third of the patients (31.9%; n = 92) had some degree of physical disability; 6.2% (n = 18) were grade 2 (10.49/1 million inhabitants) (Table 1). The factors associated with the highest risk of disability were age ≥60 years (OR, 2.83; CI, 1.56-5.12; *p<*0.001), black ethnicity/color (OR, 2.35; CI, 1.01-5.45; *p =* 0.04), and multibacillary (MB) leprosy (OR, 3.78; CI, 2.23-6.36; *p<*0.001) (Table 2).

**TABLE 2:** Factors associated with the presence of physical disabilities in new leprosy cases diagnosed in Arapiraca (Alagoas, Northeast Region, Brazil), 2008-2017 (n = 292).

Variable	Physical Disability		OR^a^	***p*-value**	CI 95%
	Grade 1 or 2	Grade 0			
	% (n)	% (n)			
**Gender**					
Male	32.1 (43)	67.9 (91)	--	--	--
Female	31.0 (49)	69.0 (109)	1.051	0.844	0.64*-*1.72
Age group*					
<60	26.8 (63)	73.2 (172)	2.828	<0.001*	1.56*-*5.12
≥60	50.9 (29)	49.1 (28)	--	--	--
**Schooling**					
<5 years	26.6 (29)	73.4 (80)	--	--	--
≥5 years	34.4 (63)	65.6 (120)	0.609	0.164	0.41*-*1.16
Race/color*					
Others	29.9 (80)	70.1 (70.1)	--	--	--
Black	50.0 (12)	50.0 (12)	2.350	0.042*	1.01*-*5.45
Classification Operational*					
Paucibacillary	18.6% (29)	81.4% (127)	--	--	--
Multibacillary	46.3% (63)	53.7% (73)	3.779	<0.001*	2.23*-*6.39

^a^ **OR**: odds ratio; **CI:** confidence interval. **(--):** reference categories. *Significant association (*p-value<*0.05).

### Temporal modeling

**FIGURE 2:**
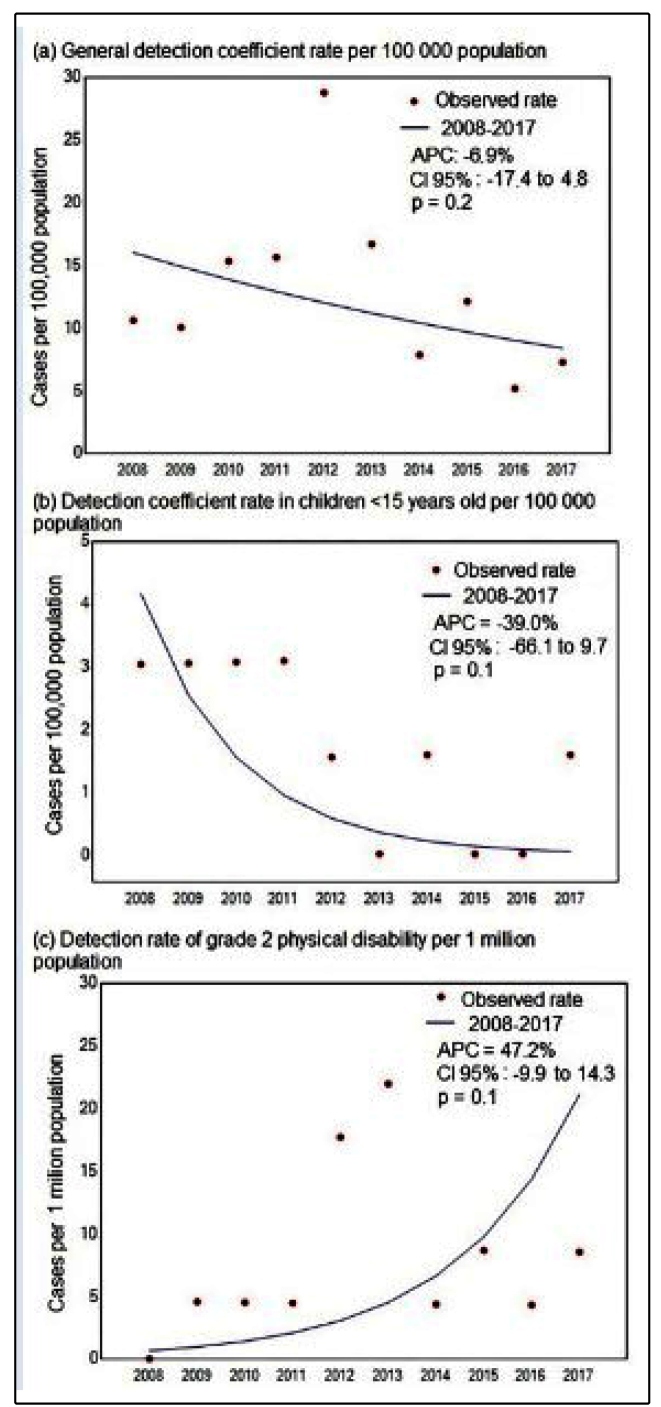
Detection coefficient of leprosy and grade 2 disability in Arapiraca. Joinpoint regression model of the detection coefficients of new cases (A) in the general population/100,000; (B) in children under 15 years old/100,000; and (C) the detection rate of grade 2 physical disability/1 million. APC: Annual Percent Change; CI: confidence interval; *p*: P-value.

The detection coefficient of new leprosy cases in the general population has changed from 10.60/100 thousand in 2008 (the beginning of the temporal series) to 7.25/100 thousand in 2017 (APC, -6.9%; CI, -17.4-4.8; *p =* 0.20; Figure 2A). The detection coefficient of leprosy in children under 15 years old, in turn, ranged from 3.03/100 thousand in 2008 to 1.59/100 thousand in 2017 (APC, -39.0%; CI, -66.1-9.7; *p =* 0.1; Figure 2B). There was also a change in the detection coefficient of grade 2 disability, which varied to 0.01/1 million in 2008 to 8.54/1 million in 2017 (APC, 47.2%; CI, -9.9-14.3; *p =* 0.1; Figure 2C). However, these trends were not statistically significant and the three indicators studied showed stationary temporal behavior, according to the joinpoint regression model.

### Spatial modeling

**FIGURE 3:**
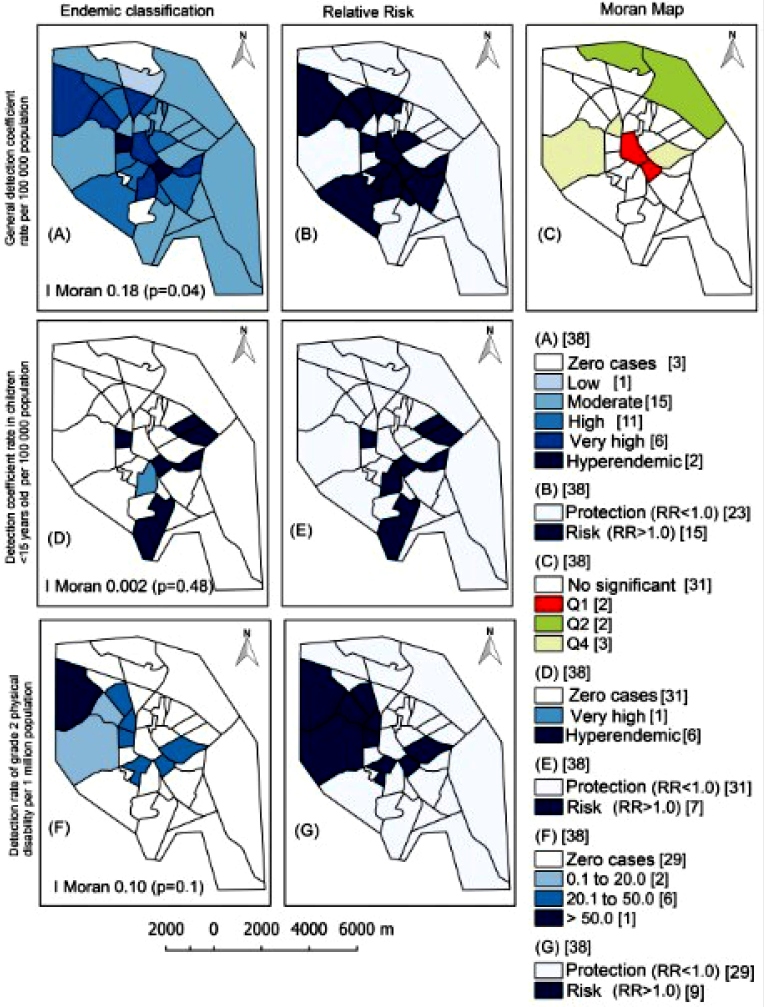
Spatial distribution of leprosy in Arapiraca (Alagoas, Northeast Region, Brazil), 2008-2017. Endemic classification, relative risk, and Moran map of leprosy detection coefficients (A-C) in the general population/100,000; (D and E) in children under 15 years old/100,000; and (F and G) of the detection rate of grade 2 physical disability/1 million. Moran scattering diagram (C) indicates whether the chance of the event occurring between neighborhoods is high (+) or low (-): Q1: (+,+), Q2: (-,-) and Q4: (-,+). The number of neighborhoods for each category is shown in square brackets. Q: Quadrant; Moran’s I: Moran’s statistic; RR: relative risk; *p*: P-value.

Of the 244 cases recorded in the urban area of Arapiraca, 36.47% (n = 89) were concentrated in four neighborhoods: Primavera, Baixão, Ouro Preto, and downtown. Eight neighborhoods (21.05%) were classified as having very high endemicity (20.00-39.99/100 thousand) or hyperendemicity (≥40 cases/100 thousand) for the general detection coefficient, particularly Baixão (47.64/100 thousand; n = 20; RR, 3.68) and Ouro Preto (40.06/100 thousand; n = 15; RR, 3.10) (Figures 3A and 3B). In the Moran Map, downtown Arapiraca and Ouro Preto were considered to be a priority as the highest risk of leprosy occurrence (Figure 3C).

In children under 15 years old, 54.54% (n = 6) were concentrated in three neighborhoods (Jardim Tropical, Itapoã, and Primavera), and six neighborhoods were considered hyperendemic (≥10 cases/100 thousand): Jardim Tropical (35.33/100 thousand; n = 2; RR, 20.66), Itapoã (32.41/100 thousand; n = 2; RR, 18.95), Guaribas (24.50/100 thousand; n = 1; RR, 14.33), Novo Horizonte (16.36/100 thousand; n = 1; RR, 9.57), Ouro Preto (12.70/100 thousand; n = 1; RR, 7.43), and Baixão (10.92/100 thousand; n = 1; RR, 6.39). Moran’s I statistic showed no spatial dependence for this indicator (Moran’s I = 0.002; *p =* 0.48) (Figures 3D and 3E).

With regard to the detection coefficient of new leprosy cases with grade 2 physical disability, seven neighborhoods had rates higher than 20.00/1 million inhabitants; Senador Nilo Coelho (92.05/1 million; n = 4; RR, 11.55), Cacimbas (30.45/1 million; n = 2; RR, 3.82), and Eldorado (29.14/1 million; n = 1; RR, 3.65) were the most prominent neighborhoods. There was also no spatial dependence for this indicator (Moran’s I = 0.10; *p =* 0.10) (Figure 3F and 3G).

## DISCUSSION

The analysis of leprosy showed nuances relevant to the sickening process, disability, and transmission chain in the municipality of Arapiraca. In the analyzed period, there was a slight predominance of women (54.1%), similar to that observed in the states of Maranhão (52.3%) and Sergipe (51.1%)^23^ ^,^ ^24^. This may reflect the higher demand for health services by the female population, as shown in other studies^25^ ^,^ ^26^ ^,^ ^27^.

On the other hand, some studies have shown that men neglect their health^28^ ^,^ ^29^. According to the MF and some studies carried out in Bahia, Pará, and Alagoas, there is a higher incidence of leprosy in men, which differs from what has been observed in our study, suggesting the existence of a higher number of hidden, chronic and transmission cases in the male population of Arapiraca^11^ ^,^ ^30^ ^,^ ^31^ ^,^ ^32^.

Regarding the age group, there was a preponderance of people who were at an economically active age. This profile is similar to that found in Minas Gerais, Maranhão, and, internationally, in Dhaka and Bangladesh^33^ ^,^ ^34^ ^,^ ^35^. Physical disabilities have been a challenge in the fight against leprosy in Brazil, often leading to temporary or permanent sick leave from work, which may result in consequences for social security in the long-term^36^ ^,^ ^37^ ^,^ ^38^ ^,^ ^39^.

Concomitantly, in our study, the risk of disability increased with age (OR, 2.83 in the elderly), corroborating a study conducted in Juazeiro, Bahia (OR, 2.20 for people over 45 years old)^40^. The higher risk of developing leprosy in the elderly is due to the gradual and inevitable changes in the aging process, with damage in dynamics and functional capacity and a lower immunological competence in this group^41^ ^,^ ^42^ ^,^ ^43^ ^,^ ^44^.

In our study, black people were about twice as likely to develop disability (OR, 2.35). This can be explained by the fact that these people have lesser access to health services, in addition to their socioeconomic vulnerability, which is in line with studies on access to health services in Brazil^45^ ^,^ ^46^ ^,^ ^47^. Thus, the analysis of components related to leprosy leads us to reflect on individual vulnerability and its consequences^48^.

One of the dimensions of social vulnerability is related to low schooling level (up to eight years of education), which results in a higher number of leprosy cases in this category (65.4%)^49^. This is also described in studies conducted on the border between Mato Grosso do Sul-Paraguay and Cuiabá, which found that 61.7% and 61.8% of the study population, respectively, were classified in this group^50^ ^,^ ^51^. In our study, there was no statistically significant association between schooling level and degree of physical disabilities, probably due to the lack of notification relating to this variable (15.1% lacking).

The clinical form of the disease and MB operational classification are other factors that may indicate a late diagnosis and a higher risk of physical disability^5^. Our study showed that people with MB leprosy are approximately four times more likely to develop this HP (OR, 3.78), corroborating a study carried out in Maranhão (OR, 1.24 for grade 1 disability; OR, 1.20 for grade 2 disability)^43^. The diagnostic delay is reinforced by the increase in new MB cases, from 26.08% in 2008 to 52.94% in 2017. Despite the higher prevalence of the dimorphic form recorded in other studies, the tuberculoid form predominance (38.7%) found in our study may be a worrisome result since it is an indication that the disease affects individuals with a more resistant immunological profile, which was also observed in Juazeiro, Bahia^23^ ^,^ ^31^ ^,^ ^40^.

Despite the slight predominance of people with PB leprosy (53.4%), the epidemiological magnitude of the endemic disease should be considered since almost half of the cases were MB, which is evidence of a process of active transmission in the community. A study conducted in Ethiopia recorded 89% of cases being MB, which also indicates a continuity of this process^52^. Another study found a predominance of MB cases (72.5%) in Maranhão from 2012 to 2015^43^. Conversely, PB cases were more prominent in Piauí, from 2011 to 2014 (51.79%), which is similar to our findings^53^.

Referral (83.2%) was the main mode of detecting leprosy cases, followed by spontaneous demand (8.2%), a predominance also observed at a national level from 2012 to 2016 and in Rio de Janeiro^11^ ^,^ ^54^. The diagnosis, treatment, and monitoring of leprosy cases are performed in the Integrated Reference Center of Arapiraca, which may justify the percentages found here. These data may indicate a deficient active search for new cases, a fact also reported in other studies^30^ ^,^ ^55^. 

This epidemiological context reinforces the persistence of the transmission chain in Arapiraca, which showed a stationary trend for the detection coefficients in the general population, in children under 15 years old, and in patients with grade 2 physical disability. This scenario might be even more serious since some studies suggest that the number of cases recorded is lower than the actual number of patients with the disease, resulting in a high hidden prevalence^7^ ^,^ ^56^. This is confirmed by some studies which have estimated that the actual number of new leprosy cases might be 6-8 times higher than that already reported^56^ ^,^ ^57^ ^,^ ^58^ ^,^ ^59^. In our temporal dynamics results, a peak in leprosy indicators (general and grade 2 physical disability) was observed between 2012 and 2013 (Figure 2A and Figure 2C). This peak can be explained by efforts to detect the disease during this period, through active search actions promoted by the Municipal Health Department, during one month, and directed at the central region of the city. 

A study in northwest Bangladesh showed that the actual prevalence of the disease was 13.1 cases/100 thousand inhabitants, compared to the recorded data (2.31 cases/100 thousand inhabitants)^58^. According to another study, 28.4% of leprosy cases were not identified by the health care system in Brazil^57^. The prevalence rate in Alagoas is less than 1/10,000, which characterizes leprosy as an eradicated disease. However, two studies published in 2019 found evidence of pseudo-elimination, in which there was a high proportion of MB cases, persistent transmission in children under 15 years old, and a high rate of people with grade 2 physical disability^7^ ^,^ ^60^.

The spatial distribution analysis showed significant heterogeneity in the neighborhoods of Arapiraca, suggesting a high hidden prevalence of leprosy and late diagnosis. This heterogeneous pattern has been observed at different territorial scales^16^ ^,^ ^32^. Several factors may be associated with this finding, such as the specific characteristics of the population, socioeconomic conditions, a neglect of personal health, and access to health services^54^.

Critical areas were identified for the three epidemiological indicators analyzed, as well as areas of hyperendemicity and relative risk of developing leprosy in Arapiraca. These cluster areas are at high risk for leprosy transmission because they favor that transmission and affect people living in poor social conditions, with no basic sanitation and a precarious socio-economic situation and housing. Reduced levels of income and education as well as factors that reflect unfavorable living conditions have been associated with an increase of up to two orders of magnitude in the incidence of leprosy^16^ ^,^ ^30^ ^,^ ^61^.

The downtown neighborhoods of Arapiraca (the historical origin of the municipality and current commercial center) had a higher number of cases, which may confirm the existence of a greater flow of people in these areas. This is also where the older population of Arapiraca is most concentrated. For these reasons, the downtown neighborhoods may also have a higher number of possible latent cases. Similar results were found in Juazeiro (Bahia, Brazil) where one of the highest density areas was located downtown.

A limiting factor of this study is the fact that it analyzes secondary data, which may be operationally influenced by the epidemiological surveillance system. However, the models used in this research allowed us to characterize leprosy in the neighborhoods of Arapiraca, leading to an understanding of the spatial and temporal aspects of the disease. Another limiting factor is the fact that the indicators were determined by combining several variables, aggregated in a single index, which may make it difficult to identify each isolated component in, and its respective contribution to, the social determination of leprosy in the studied municipality.

The analyzed epidemiological indicators revealed the complexity of the process for leprosy development. The spatial analysis of the distribution of leprosy cases in Arapiraca showed a heterogeneous distribution in the neighborhoods, concentrated in Primavera, Baixão, Ouro Preto, and Downtown. In addition, these spatial and temporal studies are extremely relevant to assist with planning, monitoring, and guiding interventions in the municipality. The stationary trend for leprosy indicators, the high incidence in the city's neighborhoods and the association of grade 2 physical disability with the age group and operational classification factors help to reveal the underdiagnosis, late diagnosis, and continued transmission of leprosy in Arapiraca.
